# Voluntary Wheel Running Partially Attenuates Early Life Stress-Induced Neuroimmune Measures in the Dura and Evoked Migraine-Like Behaviors in Female Mice

**DOI:** 10.3389/fphys.2021.665732

**Published:** 2021-05-28

**Authors:** Olivia C. Eller, Xiaofang Yang, Isabella M. Fuentes, Angela N. Pierce, Brittni M. Jones, Aaron D. Brake, Ruipeng Wang, Gregory Dussor, Julie A. Christianson

**Affiliations:** ^1^Department of Anatomy and Cell Biology, University of Kansas Medical Center, Kansas City, KS, United States; ^2^Department of Physiology, Kansas City University of Medicine and Biosciences, Joplin, MO, United States; ^3^School of Behavioral and Brain Sciences, The University of Texas at Dallas, Dallas, TX, United States; ^4^Department of Anesthesiology, University of Kansas Medical Center, Kansas City, KS, United States

**Keywords:** mast cell, migraine, hypothalamic-pituitary-adrenal axis, calcitonin gene-related peptide, mouse grimace scale, photophobia, voluntary wheel running

## Abstract

Migraine is a complex neurological disorder that affects three times more women than men and can be triggered by endogenous and exogenous factors. Stress is a common migraine trigger and exposure to early life stress increases the likelihood of developing chronic pain disorders later in life. Here, we used our neonatal maternal separation (NMS) model of early life stress to investigate whether female NMS mice have an increased susceptibility to evoked migraine-like behaviors and the potential therapeutic effect of voluntary wheel running. NMS was performed for 3 h/day during the first 3 weeks of life and initial observations were made at 12 weeks of age after voluntary wheel running (Exercise, -Ex) or sedentary behavior (-Sed) for 4 weeks. Mast cell degranulation rates were significantly higher in dura mater from NMS-Sed mice, compared to either naïve-Sed or NMS-Ex mice. Protease activated receptor 2 (PAR2) protein levels in the dura were significantly increased in NMS mice and a significant interaction of NMS and exercise was observed for transient receptor potential ankyrin 1 (TRPA1) protein levels in the dura. Behavioral assessments were performed on adult (>8 weeks of age) naïve and NMS mice that received free access to a running wheel beginning at 4 weeks of age. Facial grimace, paw mechanical withdrawal threshold, and light aversion were measured following direct application of inflammatory soup (IS) onto the dura or intraperitoneal (IP) nitroglycerin (NTG) injection. Dural IS resulted in a significant decrease in forepaw withdrawal threshold in all groups of mice, while exercise significantly increased grimace score across all groups. NTG significantly increased grimace score, particularly in exercised mice. A significant effect of NMS and a significant interaction effect of exercise and NMS were observed on hindpaw sensitivity following NTG injection. Significant light aversion was observed in NMS mice, regardless of exercise, following NTG. Finally, exercise significantly reduced calcitonin gene-related peptide (CGRP) protein level in the dura of NMS and naïve mice. Taken together, these findings suggest that while voluntary wheel running improved some measures in NMS mice that have been associated with increased migraine susceptibility, behavioral outcomes were not impacted or even worsened by exercise.

## Introduction

Migraine is a neurological disorder that presents as throbbing cranial pain, sensitivity to light (photophobia) and sound (phonophobia), nausea, fatigue, irritability, muscle tenderness, and cutaneous allodynia (Dodick, 2018). Migraine is often triggered or exacerbated by stress (Spierings et al., 2001; Nash and Thebarge, 2006) and stress experienced early in life is associated with increased susceptibility to developing migraine in adulthood (Felitti et al., 1998; Anda et al., 2010; Brennenstuhl and Fuller-Thomson, 2015). As such, abnormal activity of the hypothalamic-pituitary-adrenal (HPA) axis, the main stress response system of the body, has been detected in chronic migraine patients (Patacchioli et al., 2006; Rainero et al., 2006; Juhasz et al., 2007). Administration of nitroglycerin (NTG), a known migraine trigger, significantly increased plasma cortisol levels in migraineurs, compared to healthy controls (Juhasz et al., 2007). Mast cells, which are innate immune cells found in close approximation to sensory nerve endings in the dura, express receptors for corticotropin-releasing factor (CRF), the main stress hormone released by the hypothalamus (Theoharides et al., 2004). Activation of mast cells and their subsequent release of histamine, tryptase, and cytokines, can sensitize dural nociceptors and evoke their release of vasoactive neuropeptides, such as calcitonin gene-related peptide (CGRP), which has been shown to contribute to migraine pathology (Theoharides et al., 2005).

Animal studies have shown evidence of stress worsening migraine-like behavior and outcomes. Chronic restraint stress induced thermal hyperalgesia that was further exacerbated by NTG injection (Costa et al., 2005), while a 14-day social defeat stress (SDS) paradigm or a 40-day chronic variable stress (CVS) paradigm had similar impacts on increasing hindpaw mechanical allodynia following NTG injection (Kaufmann and Brennan, 2018). Repeated restraint stress induced facial mechanical allodynia and increased MGS in rats and also resulted in transient hyperalgesic priming (Avona et al., 2020). Pretreatment with polyclonal antiserum to CRF significantly reduced mast cell degranulation following restraint stress (Theoharides et al., 1995) and pretreatment with a glucocorticoid receptor antagonist blocked an increase in cortical spreading depression in a transgenic mouse model of familial hemiplegic migraine following treatment with corticosterone (Shyti et al., 2015). A model of secondary traumatic stress during the neonatal period is the only published early life stress model used to study migraine, which has shown increased expression of CGRP, signal transduction proteins, and glial fibrillary acidic protein in the spinal trigeminal nucleus (Hawkins et al., 2018) and increased facial allodynia following exposure to a pungent odor (Peterson et al., 2020).

Although strenuous physical activity is a known migraine trigger, being physically inactive is associated with migraine and submaximal aerobic exercise can reduce the frequency of episodes and improve quality of life in migraineurs (Daenen et al., 2015). Exercised-based improvements in migraine are generally associated with increased serotonin and endogenous opioid levels; however, exercise-induced improvements in common comorbid mood disorders, such as anxiety and depression, can be, at least partially, attributed to normalizing output from the HPA axis (Hearing et al., 2016). Our model of early life stress in mice, using neonatal maternal separation (NMS) demonstrates urogenital hypersensitivity, increased MC degranulation in the affected organs, and reduced expression of stress-related regulatory genes in the hypothalamus and hippocampus, which is a major inhibitory regulator of the HPA axis (Pierce et al., 2014, 2016; Fuentes et al., 2017). Voluntary wheel running attenuated many of these NMS-related outcomes and increased brain-derived neurotrophic factor (BDNF) expression and neurogenesis in the hippocampus (Pierce et al., 2018; Fuentes et al., 2020). Here, we are using our NMS model to determine if early life stress exposure in mice can increase susceptibility to evoked migraine-like behaviors. We are also testing the impact of voluntary wheel running on molecular and behavioral outcomes related to migraine. Although migraine can affect both sexes, we carried out these studies in female mice as women comprise the majority of migraineurs (Burch et al., 2018).

## Materials and Methods

### Animals

All experiments were performed on female C57Bl/6 mice (Charles River, Wilmington, MA, United States) born and housed in the Research Support Facility at the University of Kansas Medical Center. Mice were housed at 22°C on a 12-h light cycle (600–1800 h) and received water and food *ad libitum*. All research was approved by the University of Kansas Medical Center Institutional Animal Care and Use Committee in compliance with the National Institute of Health Guide for the Care and Use of Laboratory Animals. No attempts were made to control or track the estrus cycle of the mice to avoid potentially confounding stressors and our previous studies have shown no impact of cycle stage on other outcomes related to NMS exposure (Pierce et al., 2014, 2016, 2018).

### Neonatal Maternal Separation

**Figure 1:**
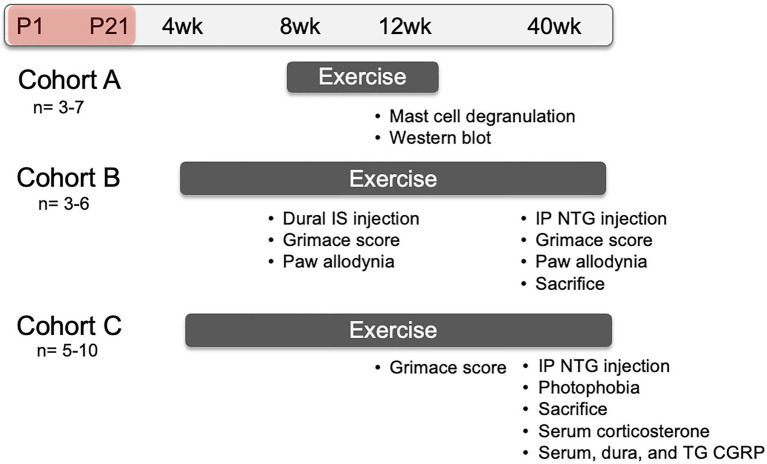
Representative timeline of evoked migraine experiments and outcome measures. All groups consisted of mice that underwent neonatal maternal separation (NMS) from postnatal day (P) 1 to 21 (depicted as the red shaded period) and non-separated, naïve mice. Mice in Cohort A were singly-housed with running wheels at 8 weeks of age and sacrificed at 12 weeks of age. Dura mater was collected to evaluate mast cell (MC) characteristics and measure protein level using Western Blot. Mice in Cohort B were pair-housed with running wheels at 4 weeks of age. At 8 weeks of age, they received either inflammatory soup (IS) or saline applied to the dura mater and, 1 h later, mouse grimace score (MGS) was measured over 1 h. Forepaw mechanical withdrawal threshold was measured immediately after cessation of MGS. At 40 weeks of age, Cohort B mice received a single intraperitoneal (IP) injection of nitroglycerin (NTG) and 30 min later were assessed for MGS over 1 h, immediately followed by hindpaw mechanical withdrawal threshold. Mice in Cohort C were also pair-housed with running wheels at 4 weeks of age and assessed for MGS, without dural stimulation of NTG, at 12 weeks of age. The mice were given an IP NTG injection at 40 weeks, followed 30 min later by photophobia testing and sacrifice. The level of corticosterone was measured in the serum and the level of calcitonin gene-related peptide (CGRP) was measured in the serum, dura, and trigeminal ganglia (TG).

Pregnant C57Bl/6 dams at 14–16 days gestation were ordered from Charles River and housed at the Department of Laboratory Animal Resources at the University of Kansas Medical Center. Litters were divided equally into NMS and naïve groups. NMS pups were removed as whole litters from their home cage for 180 min (11 am–2 pm) daily beginning at postnatal day 1 (P1) until P21. During separation, pups were placed in a clean glass beaker with bedding from their home cage. The beaker was placed in an incubator maintained at 33°C and 50% humidity. Naïve mice remained undisturbed in their home cage except for normal animal husbandry. Entire litters were designated as naïve or NMS to avoid excess stress exposure to the naïve mice. All mice were weaned on P22 and housed 2–5/cage with same sex litter mates and *ad libitum* access to food and water. All litters also contained male pups, which were similarly handled, but not investigated in this study. Three different cohorts of only female mice were used in the following experiments and are depicted on a timeline in Figure 1.

### Exercise

At either 4 or 8 weeks of age, NMS and naïve mice were equally divided into exercised (Ex) and sedentary (Sed) groups. Mice at 4 weeks of age were pair-housed and mice at 8 weeks of age were singly housed in a cage with free access to a stainless-steel running wheel (STARR Life Sciences Corp., Oakmont, PA, United States). Pair-housed mice had constant access to the running wheel and were observed running on the wheel simultaneously. Sed mice remained pair- or group-housed (2–5/cage) in their home cage with no access to a running wheel. Distance ran was recorded by STARR Life Sciences VitalView Activity Software version 1.1.

### Dural Mast Cell Staining

Mice were overdosed with inhaled isoflurane (<5%) and intracardially perfused with ice-cold 4% paraformaldehyde (PFA). Dura was removed from the skull and post-fixed in 4% PFA at 4°C for 1 h and cryopreserved in 30% sucrose in phosphate buffered saline (PBS). Dura was whole mounted on a glass microscope slide and stained for 10 min with 1% toluidine blue (TB) solution acidified with 1 M HCL. Slides were allowed to dry for 2 h in a 37°C oven, washed in 95% then 100% EtOH, fixed in xylene, and cover slipped. Using a light microscope (Nikon eclipse 90i, Nikon Instruments, Inc., Melville, NY, United States) 10 non-adjacent images of each dura sample were taken (QIClick digital CCD Camera, QImaging, Surrey, BC, Canada). The total number and the number of degranulated mast cells were counted in each field (800 um^2^ per field) per tissue. The percent of degranulated mast cells was quantified using the following equation: (Degranulated mast cells/total mast cells) × 100.

### Western Blots

Mice were overdosed with inhaled isoflurane (<5%) and dura was removed and flash frozen in liquid nitrogen before storage at −80°C. Dural protein was isolated using Cell Extraction Buffer containing Halt protease and phosphatase inhibitors (Thermo-Fisher Scientific, Waltham, MA, United States) and Na_3_VO_4_. Protein concentrations were determined using a Pierce BCA assay (Thermo-Fisher Scientific, Waltham, MA, United States). Samples were reduced by heating to 95°C in the presence of 2-mercaptoethanol, subjected to SDS-PAGE (Criterion 4–12% Bis-Tris gels; Bio-rad laboratories), and transferred to Nitrocellulose transfer membrane (Whatman GmbH, Dassel, Germany) by Criterion Blotter wet transfer (Bio-Rad). The membranes were blocked for 1 h in 5% milk in Tris-buffered saline with Tween-20 (TBST) then incubated overnight with protease-activated receptor 2 (PAR2; 1:1000; Abcam), transient receptor potential ankyrin 1 (TRPA1;1:1000; Aviva) or glyceraldehyde 3-phosphate dehydrogenase (GAPDH;1:2000; Cell Signaling) antisera. Membranes were washed with TBST and incubated for 1 h with anti-rabbit secondary antibody (1:10,000; Cell Signaling). Densitometry was performed using Quantity One 4.6.9 software (Bio-Rad Laboratories). PAR2 and TRPA1 protein levels were normalized to GAPDH.

### Dural Injection

Under inhaled isoflurane a modified cannula injector (Plastics One) was inserted at the lambdoidal suture without penetrating the dura (Burgos-Vega et al., 2019). About 10 ml of inflammatory soup (IS; 1 mM histamine, 1 mM 5-hydroxytryptamine, 1 mM bradykinin, and 0.1 mM prostaglandin E2 in PBS) or saline was slowly dispersed onto the dura and the injector was removed.

### NTG Injection

Mice received an intraperitoneal (IP) NTG or saline injection at 10 mg/kg.

### Mouse Grimace Scale

The mouse grimace scale (MGS) is a measure of facial expressions indicating spontaneous pain-like behavior in mice. It is a set of five behaviors: orbital tightening, nose bulge, cheek bulge, ear position, and whisker change. Each facial expression is rated as “not present (scored 0), moderate (scored 1), or severe (scored 2)” and then an overall score is assigned (Langford et al., 2010). Mice were placed on top of a wire mesh screen elevated 55 cm above a table and enclosed under an over-turned 500 ml glass beaker. Behavior was video recorded for 1 h. Facial screen shots were then taken from the videos every 5 min. Photographs of each mouse were randomized and grimace score was assigned to each picture according to a modified version of the MGS established by Langford et al. (2010). Only orbital tightening and ear position were scored due to the difficulty of scoring the other features (nose bulge, cheek bulge, and whisker change) on a C57Bl/6 mouse. An average grimace score from two blinded investigators was quantified for each mouse.

### Paw Sensitivity

Before testing for paw mechanical sensitivity, mice were acclimated to a sound-proof room and the testing table for 2 days before the day of testing. This acclimation consisted of 30 min within the sound-proof room followed by 30 min inside a clear plastic chamber (11cm × 5cm × 3.5 cm) on a wire mesh screen elevated 55 cm above a table. Paw mechanical withdrawal threshold was measured using a standard set of graded von Frey monofilaments (1.65, 2.36, 3.22, 3.61, 4.08, 4.31, 4.74 g; Stoelting, Wood Dale, IL, United States) following the up-down method. The 3.22 g monofilament was used to apply force to one paw. If there was no response, the next larger grade of monofilament was used on the next round of application. If there was a positive response (e.g., raising of the paw from the table, licking paw), the next smaller grade of monofilament was used on the next round of application. After the first positive response, the up-down method was continued on alternating paws for four more applications with a minimum of five or a maximum of nine applications. The withdrawal threshold of each mouse was then quantified as a 50% g threshold for each mouse (Chaplan et al., 1994).

### Light Aversion Behavior

A modified force plate actimeter was used to assess light aversion behavior. An opaque insert with an opening in the middle was placed in the center of the box on the actimeter. This allowed the mice to move freely between both sides. The light side of the chamber was equipped with lights affixed to the ceiling and controlled by a single dimmer with low, medium, and high settings. The high setting was used and the intensity on the light side was 950 +/− 20 lux, while the intensity of the light on the dark side was less than 5 lux. The light level in the home cage was 170 +/− 20 lux. Two mice could be tested simultaneously in a 20 min session with a maximum of 15 mice tested in 1 day between 0800 and 1200 h, during the light cycle. Mice were tested 6 and 3 days before the day of NTG treatment to establish a baseline. Mice were always acclimated to the room for 1 h before testing. During the testing period, mice were placed in the lit compartment facing away from the opening and allowed to freely move between the light and dark compartments for 20 min. Movement was recorded using FPARun software (Bioanalytical Systems Inc. West Layfette, IN, United States).

### Enzyme-Linked Immunosorbent Assay

Immediately after light aversion testing, mice were overdosed with inhaled isoflurane (>5%) and trunk blood, dura, and TG were collected. Blood was allowed to clot for 1 h on ice and centrifuged at 10,000 rpm for 10 min. Serum was then collected and stored at −20°C until analysis. Dura and TG were immediately frozen in liquid nitrogen and stored at −80°C until total protein was isolated using sonication in RIPA buffer containing protease and phosphatase inhibitors. Serum corticosterone was quantified using an ELISA kit according to the manufacturer’s instructions (ALPCO, Salem, NH, United States). Serum, dura, and TG CGRP were also quantified using an ELISA kit according to the manufacturer’s instructions (MyBioSource, San Diego, CA, United States).

### Statistical Analysis

Calculations of the measurements described above were made in Excel (Microsoft, Redmond, WA, United States) and statistical analyses were performed using GraphPad Prism 8 (GraphPad, La Jolla, CA) or IBM SPSS Statistics 26 (IBM Corporation, Armonk, NY, United States). Differences between groups were determined by non-repeated or repeated-measure mixed-model ANOVA and Fisher’s least significant difference (LSD) posttest, as indicated in the figure legends. Statistical significance was set at *p* < 0.05.

## Results

### Dural Mast Cell Characteristics

**Figure 2:**
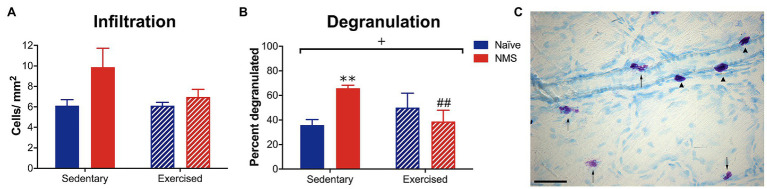
Dura mater was stained with toluidine blue (TB) to visualize mast cells and the total number and percent of degranulated mast cells were quantified in sedentary (Sed) or exercised (Ex) naïve and NMS mice. **(A)** NMS-Sed mice had a non-significant increase in the number of mast cells in the dura compared to naïve-Sed and NMS-Ex mice. **(B)** There was a significant NMS/exercise interaction on the percent of degranulated dural mast cells. NMS-Sed mice had a significantly greater percent of degranulated dura mast cells compared to naïve-Sed mice. This effect was attenuated by exercise, as NMS-Ex mice had a significantly lower percent of degranulated mast cells compared to NMS-Sed mice. **(C)** Representative image of dura mater from an NMS-Sed mouse stained with TB. Arrow heads indicate intact mast cells and arrows indicate degranulated or activated mast cells. Scale bar in C equals 25 μm. Bracket indicates a significant NMS/exercise interaction (^+^*p* < 0.05), two-way ANOVA; **p < 0.01 vs. naïve, ##*p* < 0.01 vs. sedentary, Fisher’s LSD posttest. *n* = 3–6.

Initial observations were made in naïve and NMS mice that were caged under sedentary conditions (-Sed) or received free access to a running wheel from 8 to 12 weeks of age (−Ex). During this time, naïve and NMS mice ran similar distances per week (naïve: 7.4 km ± 0.93; NMS: 8.0 km ± 0.72; *p* = 0.77, mixed-effects model). Mast cells in the dura were visualized using toluidine blue and the total number and percent that were degranulated and calculated (Figure 2). There was a non-significant increase in the number of mast cells counted in the NMS-Sed dura (Figure 2A). However, a significant overall NMS/exercise interaction effect was observed on dural mast cell degranulation, such that NMS-Sed mice had a significantly higher degranulation rate compared to either naïve-Sed or NMS-Ex mice (Figure 2B).

### Protease Activated-Receptor 2 and Transient Receptor Potential Ankyrin 1 Protein Levels in the Dura

**Figure 3:**
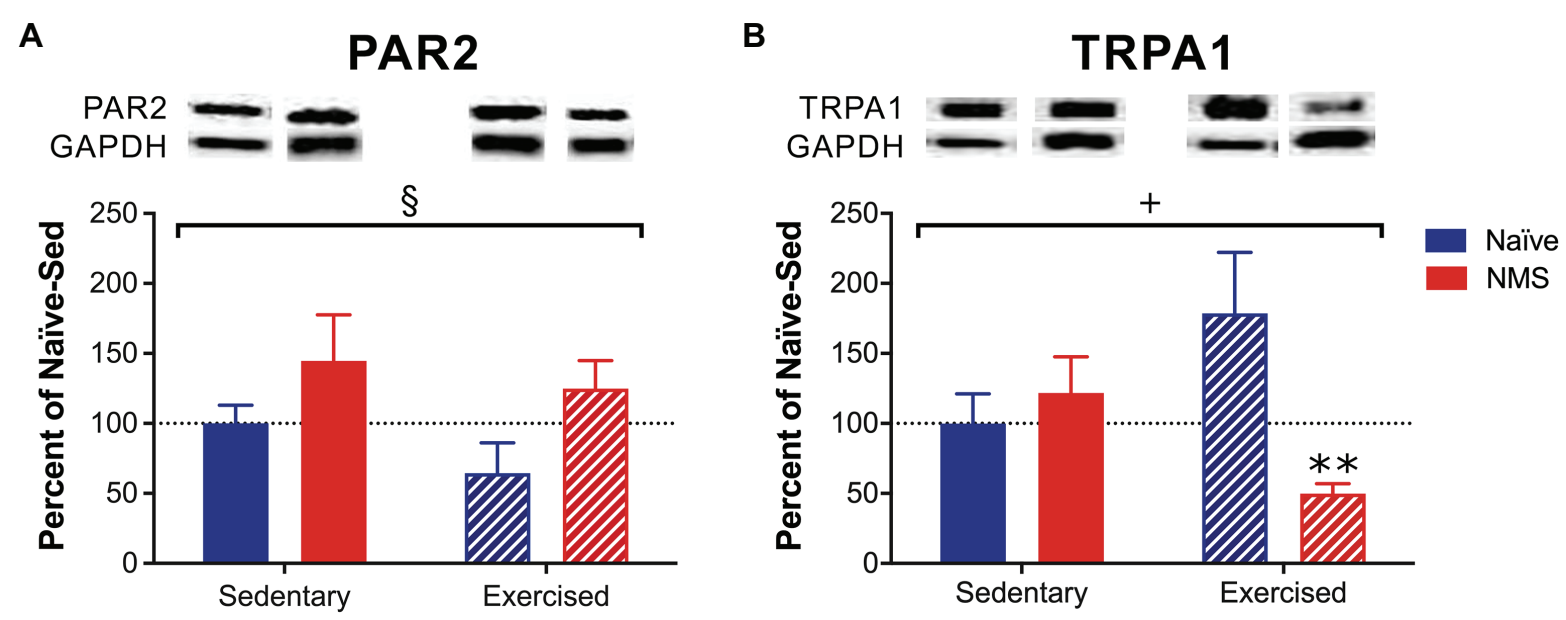
Protein levels of protease-activated receptor 2 (PAR2) and transient receptor potential ankyrin 1 (TRPA1) were measured in the dura of Sed and Ex naïve and NMS mice using Western blot. **(A)** There was a significant impact of NMS on dural PAR2 protein levels. **(B)** A significant interaction effect of NMS and exercise was observed on dural TRPA1 protein levels. NMS-Ex mice had significantly lower TRPA1 protein levels compared to naïve-Ex mice. Bracket indicates a significant effect of NMS (§*p* < 0.05) or a NMS/exercise interaction (+*p* < 0.05), two-way ANOVA; **p < 0.01 vs. naïve, Fisher’s LSD posttest. *n* = 5–7.

The protein levels of PAR2 and TRPA1 in the dura from sedentary and exercised naïve and NMS mice were measured using Western blot (Figure 3; Supplementary Figure 1). NMS significantly increased PAR2 protein levels in the dura (Figure 3A) with no significant impact of exercise. A significant interaction effect of NMS and exercise was observed for TRPA1 protein levels in the dura with NMS-Ex mice having significantly lower TRPA1 levels compared to naïve-Ex mice (Figure 3B).

### Mouse Grimace Score and Forepaw Mechanical Withdrawal Thresholds After Direct Dural Application of Inflammatory Soup

**Figure 4:**
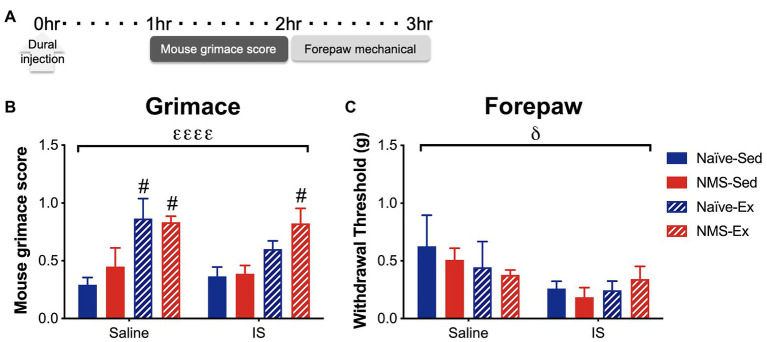
Inflammatory soup or saline was applied to the dura through a modified injection cannula of Sed or Ex naïve and NMS mice. **(A)** MGS was measured every 5 min for 1 h beginning 1 h after dural injection. Forepaw mechanical thresholds were measured immediately after. **(B)** An overall significant effect of exercise was observed on MGS, such that naïve-Ex-Saline, NMS-Ex-Saline, and NMS-Ex-IS mice all had significantly higher MGS compared to their sedentary counterparts. **(C)** A significant overall effect of IS was observed on forepaw mechanical withdrawal threshold. Bracket indicates a significant effect of exercise (εεεε*p* < 0.0001) or IS (δ*p* < 0.05), three-way ANOVA; #*p*<0.05 vs. sedentary, Fisher’s LSD posttest. *n* = 4–6.

Pair-housed mice that had access to running wheels beginning at 4 weeks-of-age were used for the remainder of the study. As previously reported (Pierce et al., 2018; Fuentes et al., 2020), we did observe a slight, but non-significant decrease in weekly running distance in NMS mice compared to naïve (naïve: 7.2 km ± 0.49; NMS: 6.1 km ± 0.43, *p* = 0.13, two-way RM ANOVA). IS or saline was applied directly to the dura, *via* a modified canula, in sedentary and exercised naïve and NMS mice. Around 1 h later, mice were evaluated for mouse grimace score (MGS), followed by forepaw mechanical sensitivity (Figure 4). An overall significant effect of exercise was observed on MGS score, such that the MGS score in naïve-Ex-Saline, NMS-Ex-Saline, and NMS-Ex-IS mice was significantly higher compared to their sedentary counterparts (Figure 4B). Dural application of IS significantly lowered forepaw withdrawal thresholds across all groups (Figure 4C).

### Mouse Grimace Score and Hindpaw Mechanical Withdrawal Threshold Following Nitroglycerin Injection

**Figure 5:**
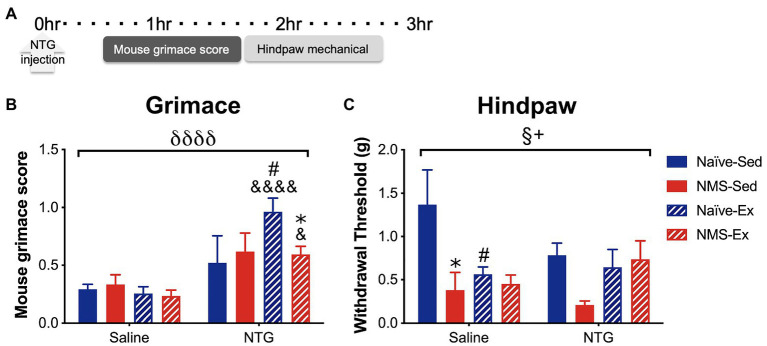
Mouse grimace score and hindpaw mechanical withdrawal threshold was measured in naïve and NMS mice that were Sed or Ex following an intraperitoneal injection of saline or NTG. **(A)** MGS was measured every 5 min over 1 h, beginning 30 min after NTG injection. Hindpaw mechanical thresholds were measured immediately afterward. **(B)** A significant overall effect of NTG was observed on MGS. Naïve-Ex-NTG mice had a significantly higher MGS then naïve-Sed-NTG and naïve-Sed-Saline mice. NMS-Ex-NTG mice had a significantly lower MGS compared to naïve-Ex-NTG mice and a significantly higher MGS than NMS-Ex-Saline mice. **(C)** A significant overall effect of NMS and a NMS/exercise interaction was observed on hind paw mechanical withdrawal threshold. NMS-Sed-Saline mice and naïve-Ex-Saline had significantly lower hind paw withdrawal thresholds compared to naïve-Sed-Saline mice. A trend toward a decreased withdrawal threshold was observed in NMS-Sed-NTG mice compared to naïve-Sed-NTG (*p* = 0.067) and NMS-Ex-NTG mice (*p* = 0.071). Bracket indicates a significant effect of NTG (δδδδ *p* < 0.0001), NMS (§ *p* < 0.05), or a NMS/exercise interaction (+*p* < 0.05), three-way ANOVA; #*p* < 0.05 vs. sedentary, &, &&&&*p* < 0.05, 0.0001 vs. saline, *p < 0.05 vs. naïve, Fisher’s LSD posttest. *n* = 3–5.

Sedentary and exercised naïve and NMS mice received an intraperitoneal (i.p.) injection of either saline or NTG (10 mg/kg) and were assessed for MGS 30 min later, followed by hindpaw mechanical sensitivity (Figure 5). NTG significantly increased MGS across all groups, specifically in naïve-Ex-NTG mice, which had a significantly higher MGS score compared to naïve-Sed-NTG, naïve-Ex-saline, and NMS-Ex-NTG mice (Figure 5B). NMS-Ex-NTG mice also had a significantly higher MGS score compared to NMS-Ex-saline mice (Figure 5B). A significant overall effect of NMS and a NMS/exercise interaction was observed on hindpaw withdrawal thresholds (Figure 5C). Naïve-Sed-saline mice had a significantly higher withdrawal threshold than naïve-Ex-saline and NMS-Sed-saline mice (Figure 5C). Although, there were no significant differences between the NTG groups, the NMS-Sed-NTG mice had a lower withdrawal threshold than naïve-Sed-NTG (*p* = 0.067) and NMS-Ex-NTG mice (*p* = 0.071). It should also be noted that our lack of significance could be due to the low animals/group we had in this set of behavioral experiments.

### Mouse Grimace Score in Mice With No Dural Stimulation

**Figure 6:**
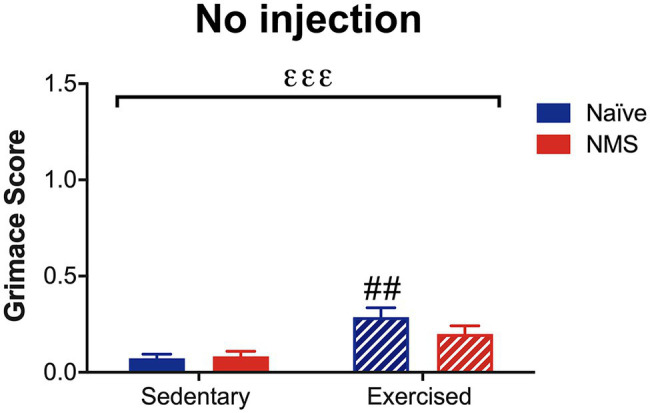
Mouse grimace score was measured in Sed and Ex naïve and NMS mice that were briefly anesthetized and allowed to recover for 1 h. There was a significant overall effect of exercise (εεε *p* < 0.001; two-way ANOVA). Naïve-Ex had a significantly greater MGS compared to naïve-Sed mice (## *p* < 0.01; Fisher’s LSD posttest). *n* = 4–5.

Due to the consistent observation that exercised mice had increased MGS scores after dural application of either IS or saline and after NTG, the next group of mice was assessed for MGS following light isoflurane anesthesia. In the absence of dural stimulation or NTG administration, a significant overall effect of exercise on MGS score was observed (Figure 6). Naïve-Ex mice, in particular, had a significantly higher MGS score compared to naïve-Sed mice and NMS-Ex mice trended toward an increase in MGS score compared to NMS-Sed mice (*p* = 0.0601).

### Photophobia-Like Behavior Following Nitroglycerin Injection

**Figure 7:**
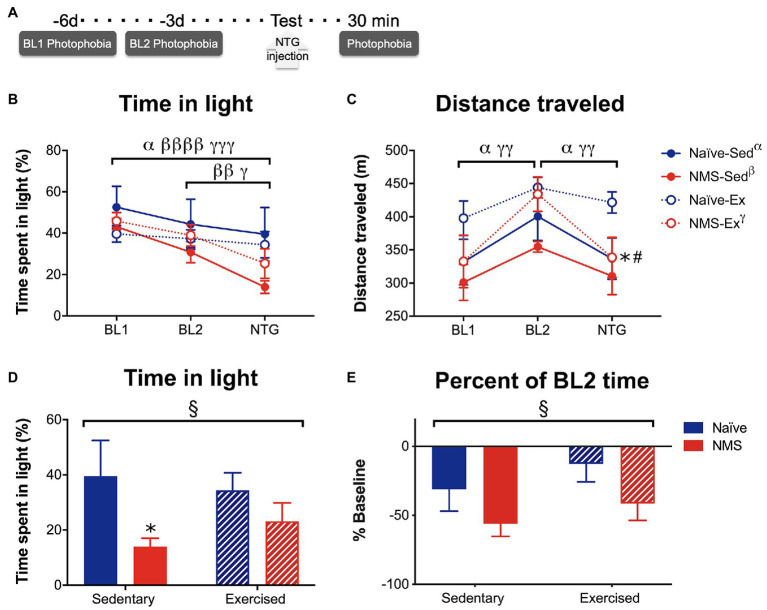
Photophobia-like behavior was measured in Sed and Ex naïve and NMS mice. **(A)** Light aversion behavior was measured 6 [baseline day 1 (BL1)] and 3 [baseline day 2 (BL2)] days prior to testing day and 30 min after an intraperitoneal NTG injection. **(B)** The percent of time spent on the light side of a light/dark box was quantified at each time point. A significant overall effect of time (*p* < 0.0001) and a NMS/time interaction (*p* < 0.05) was observed on the time spent in the light. Between BL1 and the treatment day, naïve-Sed, NMS-Sed, and NMS-Ex mice significantly decreased their time spent in the light side. Both NMS-Sed and NMS-Ex mice significantly decreased their time spent in the light between BL2 and NTG. **(C)** Total distance traveled was measured during the testing period and there was a significant overall effect of time across all groups (*p* < 0.0001). Naïve-Sed and NMS-Ex mice displayed significant changes in their distance traveled from BL1 to BL2 and from BL2 to NTG. On testing day, naïve-Ex mice traveled a significantly farther distance compared to either naïve-Sed or NMS-Ex mice. **(D)** Comparisons between groups revealed a significant impact of NMS on time in the light side following NTG treatment. NMS-Sed mice spent significantly less time in the light compared to naïve-Sed mice. **(E)** Likewise, when calculated as a percent of BL2 time spent in the light, there was a significant overall effect of NMS. Brackets indicate significant within-group differences between time points (three-way RM ANOVA, α naïve-Sed, β NMS-Sed, γ naïve-Ex, *p* < 0.05, 0.01, 0.0001, Fisher’s LSD posttest, **A,B**) or NMS (§*p* < 0.05) two-way ANOVA. *p < 0.05 vs. naïve, #*p* < 0.05 vs. sedentary, Fisher’s LSD posttest. *n* = 10.

Photophobia-like behaviors were measured over 20 min, while mice were in a light/dark box on a force place actimeter. Measurements took place on days 6 [baseline day 1 (BL1)] and 3 [baseline day 2 (BL2)] prior to assessment following treatment with NTG (Figure 7). Time spent in the light and distance traveled were quantified at each time point. Naïve-Sed, NMS-Sed, and NMS-Ex mice all spent significantly less time in the light on the NTG treatment day compared to BL1 (Figure 7B). Both NMS-Sed and NMS-Ex mice spent significantly less time in the light on the NTG treatment day compared to BL2 (Figure 7B). Naïve-Sed and NMS-Ex mice traveled significantly farther distances on BL2 compared to either BL1 or NTG (Figure 7C). Following NTG treatment, NMS significantly decreased the percent of time spent in the light, particularly in NMS-Sed mice, which spent significantly less time in the light compared to naïve-Sed mice (Figure 7D). Finally, NMS also significantly increased the change from BL2 in time spent in the light (Figure 7E).

### Calcitonin Gene-Related Peptide and Corticosterone Levels Following Nitroglycerin Injection

**Figure 8:**
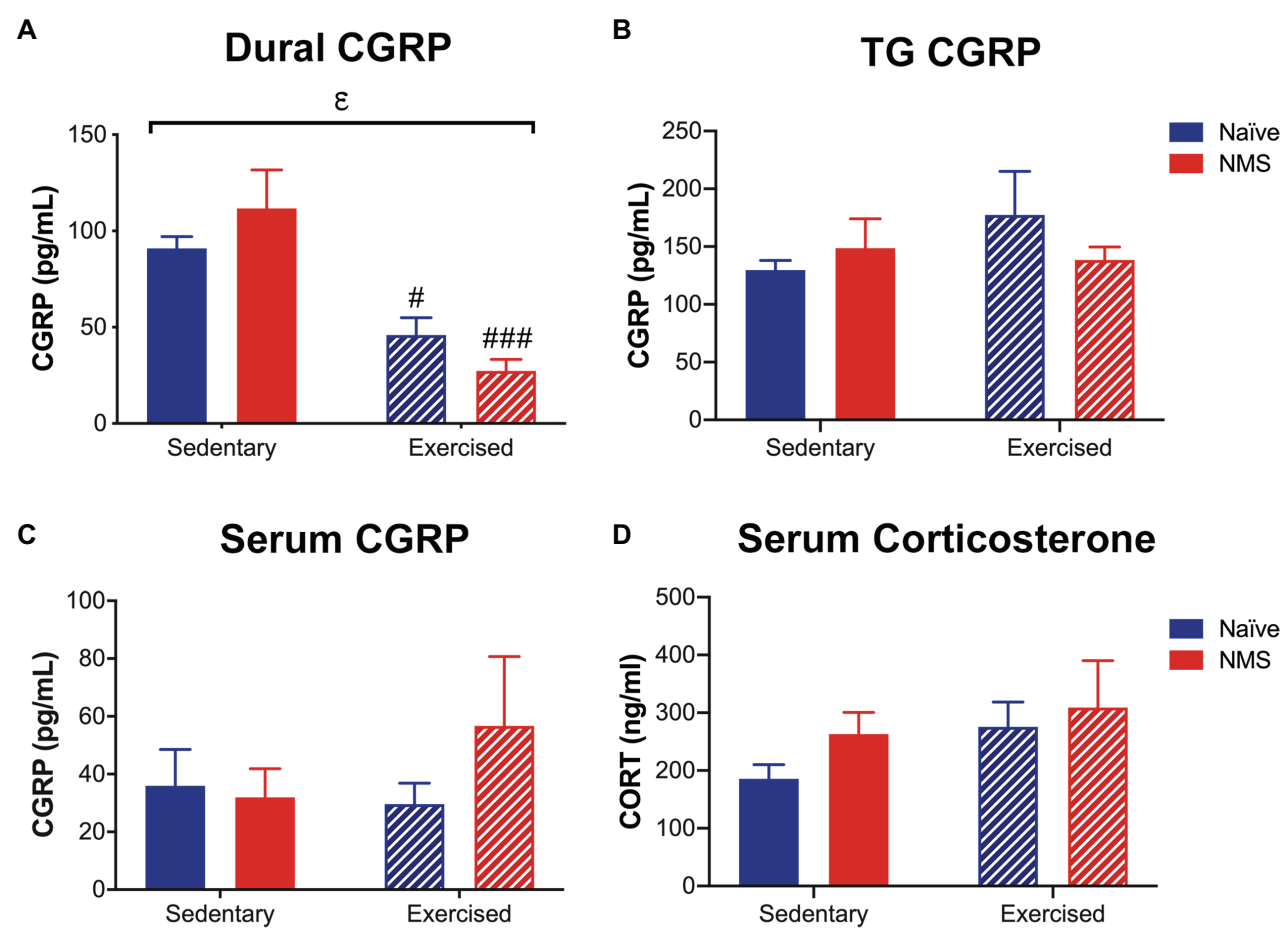
Calcitonin gene-related peptide and corticosterone levels were measured in Sed and Ex naïve and NMS mice 2 h after nitroglycerin treatment. **(A)** CGRP content in the dura was significantly decreased by exercise, such that naïve-Ex and NMS-Ex mice had significantly lower CGRP protein levels compared to their sedentary counterparts. CGRP content in the TG **(B)** and serum **(C)** was not different between groups. **(D)** Serum corticosterone levels were also not significantly different between groups. Bracket indicates a significant effect of exercise (ε*p* < 0.05) two-way ANOVA; #, ###*p* < 0.05, 0.001 vs. sedentary, Fisher’s LSD posttest. *n* = 5–6.

The protein levels of CGRP and corticosterone were measured in serum, and CGRP levels were also assessed in the dura and trigeminal ganglia (TG), 2 h after NTG injection (Figure 8). Exercise had a significant impact on decreasing CGRP levels in the dura, as both naïve-Ex and NMS-Ex mice had significantly reduced CGRP protein levels compared to their sedentary counterparts (Figure 8A). No significant differences between groups were detected for CGRP levels in the TG or serum (Figures 8B,C). Serum corticosterone levels were also not significantly different between groups (Figure 8D).

## Discussion

Migraine is a debilitating neurological disorder that affects 9.7% of males and 20.7% of females in the United States (Burch et al., 2018). It is a complicated condition with many symptoms including headache, photophobia, phonophobia, widespread allodynia, and nausea. Migraine can be triggered by both endogenous and exogenous factors (Johnson and Krenger, 1992; Anand et al., 2012), making it difficult to understand and treat. One trigger of migraine is stress and early life stress is associated with the development of migraine in adulthood (Felitti et al., 1998; Anda et al., 2010; Brennenstuhl and Fuller-Thomson, 2015). Previous preclinical and clinical research on migraine has led to discoveries of pharmacological migraine treatments that show some success, but there are often harmful off-target side effects associated with these drugs. Therefore, it is important to develop safer therapeutic interventions for migraine. Several groups have studied exercise intervention in migraineurs and found positive benefits such as increased quality of life, less frequent migraine attacks, and lower symptom intensity (Varkey et al., 2009, 2011; Darabaneanu et al., 2011). In the present study, we used a mouse model of early life stress, NMS, to determine if NMS mice displayed molecular and behavioral evidence of increased susceptibility to evoked-migraine. We also used voluntary wheel running as a non-pharmacological intervention to study if exercise had any influence on these measures.

Although the underlying pathophysiology of migraine has yet to be established, recent research has focused on the prominent role the trigeminovascular system plays in this neurological disorder (Iyengar et al., 2019). This system includes neuronal cell bodies that reside in the TG and project peripherally through the trigeminal nerve, to tissues including the dura mater, and centrally to the spinal trigeminal nucleus (Noseda and Burstein, 2013). The dura is highly innervated with nociceptive unmyelinated C-fibers and thinly myelinated Aδ fibers projecting from the ophthalmic division of the trigeminal nerve and contains many mast cells (Uddman et al., 1985; Moskowitz et al., 1988; Liu et al., 2004). Dural mast cells can become activated by several molecules including CGRP and substance P (Johnson and Krenger, 1992; Anand et al., 2012). These cells are also highly responsive to activation of the HPA axis, as they express five isoforms of the CRF_1_ receptor, a single isoform of the CRF_2_ receptor, and contain one of the largest peripheral stores of CRF (Theoharides and Cochrane, 2004). This could explain why stress is a common trigger of migraine; increased peripheral CRF release during a stressful event results in dural mast cell activation and the subsequent release of inflammatory cytokines causing a hypersensitivity reaction (Johnson and Krenger, 1992; Anand et al., 2012) that leads to migraine pain. In support of this, we found that NMS-Sed mice displayed a significant increase in the percent of degranulated mast cells in the dura compared to naïve-Sed mice. Similarly, Theoharides et al. (1995) showed rats subjected to restraint stress had increased dural mast cell degranulation, but treatment with polyclonal antiserum to CRF reduced this effect. We also found that exercise normalized the NMS-induced increase in mast cell degranulation. This effect could be due to exercise lowering susceptibility to stress, which has been demonstrated in a preclinical rodent model of uncontrollable tail shock (Greenwood and Fleshner, 2011) and is also seen in clinical research focused on exercise interventions in migraineurs (Varkey et al., 2009; Darabaneanu et al., 2011).

In addition to evaluating mast cell degranulation in the dura of our mice, we also measured dural PAR2 and TRPA1 protein levels, which are receptors that are both expressed on C- and Aδ-nociceptors (Julius and Basbaum, 2001). These receptors respond to noxious thermal, chemical, and mechanical stimuli and are often co-expressed on the same neurons (Dai et al., 2007). TRPA1 has been implicated in acute and chronic pain (Andrade et al., 2012) as well as the maintenance of hypersensitive conditions (Nassini et al., 2014). Environmental irritants that activate TRPA1 have been shown to trigger migraine headaches in susceptible individuals (Kelman, 2007). PAR2 is activated by mast cell tryptase (Ossovskaya and Bunnett, 2004) and is associated with the sensitization of TRPA1 channels through several cellular mechanisms (Dai et al., 2007; Chen et al., 2011). Activation of either receptor has also been shown to evoke migraine-like behaviors in rats (Edelmayer et al., 2012; Hassler et al., 2019). We found that there was a significant overall effect of NMS increasing dural PAR2 compared to naïve mice and a NMS/exercise interaction on the level of TRPA1 such that NMS-Ex mice had significantly less dural TRPA1 than NMS-Sed mice. We have previously observed increased PAR2 and TRPA1 protein levels in the bladder of female NMS mice, which was further increased by adult stress exposure (Pierce et al., 2016), indicating that tissue-specific expression levels of these receptors may associate with increased sensitivity. While protein levels do not reflect sensitization, it will be important to understand whether the tryptase released by degranulated mast cells is directly activating PAR2 receptors on nearby sensory nerve endings, potentially sensitizing TRPA1 receptors and increasing neuronal activity and how voluntary wheel running is selectively decreasing TRPA1 levels in NMS mice.

Facial and extracephalic hypersensitivity are commonly seen during a migraine attack in migraineurs (Burstein et al., 2000, 2010; Cuadrado et al., 2008; Edelmayer et al., 2009) and after migraine-relevant stimuli in rodents (Edelmayer et al., 2009; Wieseler et al., 2010; Stucky et al., 2011). Facial allodynia is likely caused by sensitization of second order trigeminovascular neurons found in the spinal trigeminal nucleus that receive input from the dura and the periorbital skin (Burstein et al., 1998), while extracephalic allodynia is likely caused by central sensitization (Cuadrado et al., 2008; Edelmayer et al., 2009; Burstein et al., 2010). We hypothesized that NMS mice would display paw hypersensitivity after evoked migraine compared to naïve mice and that exercise would attenuate this effect. Indeed, application of dural IS significantly lowered forepaw withdrawal thresholds across all groups. Although NTG treatment did not have a significant impact on hindpaw withdrawal thresholds, there was a significant overall effect of NMS on hind paw sensitivity, and NMS-Sed mice had a lower withdrawal threshold compared to naïve-Sed mice, however, this did not reach significance. Similar to these findings, Kaufmann and Brennan (2018) found that both stressed and non-stressed rats developed hind paw mechanical hypersensitivity after NTG injection. These results suggest that stress may not be a factor in the development of IS or NTG evoked widespread hypersensitivity. However, we only measured paw sensitivity at one time point. Future work could measure withdrawal thresholds over time in NMS and naïve mice in these evoked migraine paradigms. It is possible that NMS mice could develop IS- or NTG-evoked mechanical hypersensitivity quicker than naïve mice or that it takes longer for NMS mice to return to baseline measurements. By only measuring withdrawal threshold at one time point, we could have missed significant differences throughout the time course of the IS and NTG effects.

We also assessed grimace in our mice, which is an accepted measure of spontaneous pain-like behavior (Langford et al., 2010). We hypothesized that NMS-Sed mice would have a higher MGS score compared to naïve or exercised mice. Interestingly, there was consistently an increase in MGS score in both naïve- and NMS-Ex groups after dural IS and saline application, as well as in non-injected mice that were subject to isoflurane anesthesia. This finding was surprising because in our previous studies, we found that exercise mitigated chronic urogenital pain symptoms (Pierce et al., 2018; Fuentes et al., 2020), while a higher MGS is thought to demonstrate more pain-like behavior. It is unknown what impact voluntary wheel running has on mouse facial features and how this may impact scoring, particularly in terms of eye squint and ear position. In some instances, exercise has been shown to evoke migraine in humans. However, this is usually after strenuous exercise (Massey, 1982) or if exercise is novel and therefore it is suggested that migraineurs be slowly habituated to exercise (Goadsby and Silberstein, 2013). Our exercised mice had access to running wheels for an extended amount of time before evaluating grimace, and therefore likely did not evoke a migraine attack because of novelty. One factor that could have influenced MGS is the isoflurane anesthesia that was administered prior to MGS testing in this group of mice. Miller et al. (2015) measured MGS in DBA/2 mice before and 30-min after isoflurane anesthesia and found a significant increase in MGS following anesthesia. Although, we measured MGS 1 h after isoflurane administration, it is possible that Ex mice were not able to recover as quickly from the isoflurane and therefore exhibited a higher MGS. Further support of isoflurane influencing the exercised mice MGS is that exercise did not have a significant effect on MGS in our NTG experiment, which were not anesthetized before IP administration.

Mechanical hypersensitivity and MGS are not behaviors specific to migraine; therefore, to follow up these initial studies, light aversion was measured, which is one symptom that meets the diagnostic criteria of migraine according to the International Classification of Headache Disorders (2018). Using a light/dark box placed on a force plate actimeter, similar to the method published by Rossi et al. (2016), the location of the mouse was continuously measured during each 20 min testing period in order to quantify distance traveled and the percent time spent in the light. All groups, with the exception of naïve-Ex, significantly decreased their time spent on the light side of the box from the first baseline day to the treatment day. Both NMS groups, regardless of exercise, significantly decreased their time in the light from the second baseline day to the treatment day and there was an overall significant effect of NMS on reducing the time spent in light following NTG. These data suggest that NMS increases sensitivity to light following NTG injection. Total distance traveled during the two baseline measurements and on the treatment day were also evaluated. Interestingly, naïve-Sed and NMS-Ex mice were the only groups to show a significant increase in activity at BL2 compared to either BL1 or post-NTG. Other groups have evaluated photophobia-like behavior in rodent migraine models (Recober et al., 2010; Markovics et al., 2012; Mason et al., 2017); however, to our knowledge this is the first study to combine a stress and exercise component.

There is strong evidence that CGRP is important in the initiation and maintenance of migraine pain (Wattiez et al., 2020). CGRP is expressed in many of the unmyelinated C-fibers that innervate the dura (Eftekhari et al., 2013) and as well as TG neurons (Eftekhari et al., 2010). In addition, the CGRP receptor is expressed on the Aδ fibers that innervate the dura (Eftekhari et al., 2013) and a portion of TG neurons (Eftekhari et al., 2010). Circulating CGRP has also been shown to be elevated during migraine attacks (Goadsby and Edvinsson, 1993). We measured CGRP levels in the serum, dura, and TG of our mice following NTG injection with the hypothesis that NMS mice would display higher CGRP levels, which could explain their increased photophobia-like behavior. Surprisingly, we did not find any differences in CGRP levels between our NMS and naïve groups in any tissue that we analyzed. These results imply that increased CGRP is not the cause of the increased susceptibility to the migraine-like phenotype observed in our NMS-Sed mice. However, we did observe that exercise significantly decreased CGRP levels in the dura of both groups. To our knowledge, we are the first to measure dural CGRP in stressed and exercised mice following NTG administration. But similar to our findings, Nees et al. (2016) found that treadmill exercise decreased CGRP in the spinal cord and decreased mechanical allodynia in spinally injured mice. A limitation to our study is that we only measured dural CGRP in mice that received a NTG injection. It is possible that exercise decreases CGRP in dura regardless of NTG injection. These data could explain the positive benefits of exercise seen in clinical migraine studies (Varkey et al., 2009, 2011; Darabaneanu et al., 2011) and why our NMS-ex mice were less susceptible to dural mast cell degranulation and NTG-induced photophobia-like behavior.

Serum corticosterone following IP NTG injection and light aversion assessment was also measured. Human plasma cortisol levels are increased in migraineurs after NTG injection and this increase significantly correlated with the development of migraine (Juhasz et al., 2007). Due to the fact that NMS and exercise have been shown to influence HPA axis activity, we hypothesized that the combination of NMS and NTG would evoke an increase in circulating corticosterone compared to naïve mice, and exercise would prevent this effect. However, there was no significant effect of NMS or exercise on serum corticosterone level. Although this result implies that corticosterone may not have an effect on evoked migraine-like behaviors, corticosterone level was only measured at one time point. Circulating corticosterone is known to follow a circadian rhythm, which peaks in the early evening just before the dark cycle in laboratory rodents and is lowest at the beginning of the light cycle (Barriga et al., 2001). This is opposite to the circadian rhythm of cortisol in humans, which peaks in the early hours of the morning and decreases throughout the day (Kirschbaum and Hellhammer, 1989). Migraineurs have been shown to have a greater peak cortisol level compared to control patients (Patacchioli et al., 2006). In this study, serum corticosterone was measured when it should have been in a trough. If corticosterone were to be measured during peak hours, we might find something different.

We acknowledge several limitations in our study that may impact the interpretability of our outcomes. Cage density varied between our Ex and Sed groups, which has been shown to impact mouse physiology and behavior, such as sleep and activity levels (Toth, 2015). We chose to maintain our Sed mice in group housing conditions to avoid additional stress caused by single-caging. Our group numbers were also small for some experiments, suggesting that increasing the number of mice may have strengthened the statistical outcomes. Finally, we did not consider sex as a biological variable in this study, however, we have observed significant impacts of NMS and voluntary wheel running on urogenital sensitivity in male mice (Fuentes et al., 2020), similar to outcomes observed in female NMS mice (Pierce et al., 2018), suggesting we may see similar outcomes in both sexes.

In conclusion, we found that NMS-Sed mice had increased dural mast cell degranulation compared to naïve mice, which was attenuated by voluntary wheel running. NMS-Sed mice also appeared to be more susceptible to NTG-induced photophobia-like behavior compared to naïve mice, although exercise had no impact on these behaviors. These are important findings because they highlight the usefulness of NMS as a model to study stress-induced migraine and potentially the limited effectiveness of voluntary wheel running as a therapeutic intervention. Future work is needed to determine the underlying mechanisms of NMS and exercise in this context. Our results imply that increased CGRP is likely not involved, as CGRP level was not different between naïve and NMS groups. Voluntary wheel running decreased CGRP levels and mast cell degranulation rates in the dura; however, it also increased MGS in all of our groups that were anesthetized with isoflurane. This observation calls into question the use of MGS in previously anesthetized and exercised animals.

## Data Availability Statement

The raw data supporting the conclusions of this article will be made available by the authors, without undue reservation.

## Ethics Statement

The animal study was reviewed and approved by the Institutional Animal Use and Care Committee at the University of Kansas Medical Center.

## Author Contributions

OE, AP, GD, and JC designed the research study. OE, XY, IF, AP, BJ, AB, and RW performed the experiments. OE, XY, AP, BJ, and JC analyzed the data. OE, GD, and JC wrote the manuscript. All authors contributed to the article and approved the submitted version.

### Conflict of Interest

The authors declare that the research was conducted in the absence of any commercial or financial relationships that could be construed as a potential conflict of interest.

## References

[ref1] Anand P. Singh B. Jaggi A. S. Singh N. (2012). Mast cells: an expanding pathophysiological role from allergy to other disorders. Naunyn Schmiedeberg’s Arch. Pharmacol. 385, 657–670. 10.1007/s00210-012-0757-8 22562473

[ref2] Anda R. Tietjen G. Schulman E. Felitti V. Croft J. (2010). Adverse childhood experiences and frequent headaches in adults. Headache 50, 1473–1481. 10.1111/j.1526-4610.2010.01756.x, PMID: 20958295

[ref3] Andrade E. L. Meotti F. C. Calixto J. B. (2012). TRPA1 antagonists as potential analgesic drugs. Pharmacol. Ther. 133, 189–204. 10.1016/j.pharmthera.2011.10.008, PMID: 22119554

[ref4] Avona A. Mason B. N. Lackovic J. Wajahat N. Motina M. Quigley L.. (2020). Repetitive stress in mice causes migraine-like behaviors and calcitonin gene-related peptide-dependent hyperalgesic priming to a migraine trigger. Pain 161, 2539–2550. 10.1097/j.pain.0000000000001953, PMID: 32541386 PMC7572536

[ref5] Barriga C. Martin M. I. Tabla R. Ortega E. Rodriguez A. B. (2001). Circadian rhythm of melatonin, corticosterone and phagocytosis: effect of stress. J. Pineal Res. 30, 180–187. 10.1034/j.1600-079X.2001.300307.x, PMID: 11316329

[ref6] Brennenstuhl S. Fuller-Thomson E. (2015). The painful legacy of childhood violence: migraine headaches among adult survivors of adverse childhood experiences. Headache 55, 973–983. 10.1111/head.12614, PMID: 26104222

[ref7] Burch R. Rizzoli P. Loder E. (2018). The prevalence and impact of migraine and severe headache in the United States: figures and trends from government health studies. Headache 58, 496–505. 10.1111/head.13281, PMID: 29527677

[ref8] Burgos-Vega C. C. Quigley L. D. Trevisan Dos Santos G. Yan F. Asiedu M. Jacobs B.. (2019). Non-invasive dural stimulation in mice: a novel preclinical model of migraine. Cephalalgia 39, 123–134. 10.1177/0333102418779557, PMID: 29848109 PMC6499065

[ref9] Burstein R. Jakubowski M. Garcia-Nicas E. Kainz V. Bajwa Z. Hargreaves R.. (2010). Thalamic sensitization transforms localized pain into widespread Allodynia. Ann. Neurol. 68, 81–91. 10.1002/ana.21994, PMID: 20582997 PMC2930514

[ref10] Burstein R. Yamamura H. Malick A. Strassman A. M. (1998). Chemical stimulation of the intracranial dura induces enhanced responses to facial stimulation in brain stem trigeminal neurons. J. Neurophysiol. 79, 964–982. 10.1152/jn.1998.79.2.964, PMID: 9463456

[ref11] Burstein R. Yarnitsky D. Goor-Aryeh I. Ransil B. J. Bajwa Z. H. (2000). An association between migraine and cutaneous allodynia. Ann. Neurol. 47, 614–624. 10.1002/1531-8249(200005)47:5<614::AID-ANA9>3.0.CO;2-N, PMID: 10805332

[ref12] Chaplan S. R. Bach F. W. Pogrel J. W. Chung J. M. Yaksh T. L. (1994). Quantitative assessment of tactile allodynia in the rat paw. J. Neurosci. Methods 53, 55–63. 10.1016/0165-0270(94)90144-9, PMID: 7990513

[ref13] Chen Y. Yang C. Wang Z. J. (2011). Proteinase-activated receptor 2 sensitizes transient receptor potential vanilloid 1, transient receptor potential vanilloid 4, and transient receptor potential ankyrin 1 in paclitaxel-induced neuropathic pain. Neuroscience 193, 440–451. 10.1016/j.neuroscience.2011.06.085, PMID: 21763756

[ref14] Costa A. Smeraldi A. Tassorelli C. Greco R. Nappi G. (2005). Effects of acute and chronic restraint stress on nitroglycerin-induced hyperalgesia in rats. Neurosci. Lett. 383, 7–11. 10.1016/j.neulet.2005.03.026, PMID: 15936504

[ref15] Cuadrado M. L. Young W. B. Fernandez-De-Las-Penas C. Arias J. A. Pareja J. A. (2008). Migrainous corpalgia: body pain and allodynia associated with migraine attacks. Cephalalgia 28, 87–91. 10.1111/j.1468-2982.2007.01485.x, PMID: 18021265

[ref16] Daenen L. Varkey E. Kellmann M. Nijs J. (2015). Exercise, not to exercise, or how to exercise in patients with chronic pain? Applying science to practice. Clin. J. Pain 31, 108–114. 10.1097/AJP.0000000000000099, PMID: 24662498

[ref17] Dai Y. Wang S. Tominaga M. Yamamoto S. Fukuoka T. Higashi T.. (2007). Sensitization of TRPA1 by PAR2 contributes to the sensation of inflammatory pain. J. Clin. Invest. 117, 1979–1987. 10.1172/JCI30951, PMID: 17571167 PMC1888570

[ref18] Darabaneanu S. Overath C. H. Rubin D. Luthje S. Sye W. Niederberger U.. (2011). Aerobic exercise as a therapy option for migraine: a pilot study. Int. J. Sports Med. 32, 455–460. 10.1055/s-0030-1269928, PMID: 21472632

[ref19] Dodick D. W. (2018). Migraine. Lancet 391, 1315–1330. 10.1016/S0140-6736(18)30478-1, PMID: 29523342

[ref20] Edelmayer R. M. Le L. N. Yan J. Wei X. Nassini R. Materazzi S.. (2012). Activation of TRPA1 on dural afferents: a potential mechanism of headache pain. Pain 153, 1949–1958. 10.1016/j.pain.2012.06.012, PMID: 22809691 PMC3413768

[ref21] Edelmayer R. M. Vanderah T. W. Majuta L. Zhang E. T. Fioravanti B. De Felice M.. (2009). Medullary pain facilitating neurons mediate allodynia in headache-related pain. Ann. Neurol. 65, 184–193. 10.1002/ana.21537, PMID: 19259966 PMC2772103

[ref22] Eftekhari S. Salvatore C. A. Calamari A. Kane S. A. Tajti J. Edvinsson L. (2010). Differential distribution of calcitonin gene-related peptide and its receptor components in the human trigeminal ganglion. Neuroscience 169, 683–696. 10.1016/j.neuroscience.2010.05.016, PMID: 20472035

[ref23] Eftekhari S. Warfvinge K. Blixt F. W. Edvinsson L. (2013). Differentiation of nerve fibers storing CGRP and CGRP receptors in the peripheral trigeminovascular system. J. Pain 14, 1289–1303. 10.1016/j.jpain.2013.03.010, PMID: 23958278

[ref24] Felitti V. J. Anda R. F. Nordenberg D. Williamson D. F. Spitz A. M. Edwards V.. (1998). Relationship of childhood abuse and household dysfunction to many of the leading causes of death in adults. The adverse childhood experiences (ACE) study. Am. J. Prev. Med. 14, 245–258. 10.1016/S0749-3797(98)00017-8, PMID: 9635069

[ref25] Fuentes I. M. Jones B. M. Brake A. D. Pierce A. N. Eller O. C. Supple R. M.. (2020). Voluntary wheel running improves outcomes in an early life stress-induced model of urologic chronic pelvic pain syndrome in male mice. Pain 162, 1681–1691. 10.1097/j.pain.0000000000002178, PMID: 33399417 PMC8119308

[ref26] Fuentes I. M. Pierce A. N. Di Silvestro E. R. Maloney M. O. Christianson J. A. (2017). Differential influence of early life and adult stress on urogenital sensitivity and function in male mice. Front. Syst. Neurosci. 11:97. 10.3389/fnsys.2017.00097, PMID: 29379420 PMC5771376

[ref27] Goadsby P. J. Edvinsson L. (1993). The trigeminovascular system and migraine: studies characterizing cerebrovascular and neuropeptide changes seen in humans and cats. Ann. Neurol. 33, 48–56. 10.1002/ana.410330109, PMID: 8388188

[ref28] Goadsby P. J. Silberstein S. D. (2013). Migraine triggers: harnessing the messages of clinical practice. Neurology 80, 424–425. 10.1212/WNL.0b013e31827f100c, PMID: 23345640

[ref29] Greenwood B. N. Fleshner M. (2011). Exercise, stress resistance, and central serotonergic systems. Exerc. Sport Sci. Rev. 39, 140–149. 10.1097/JES.0b013e31821f7e45, PMID: 21508844 PMC4303035

[ref30] Hassler S. N. Ahmad F. B. Burgos-Vega C. C. Boitano S. Vagner J. Price T. J.. (2019). Protease activated receptor 2 (PAR2) activation causes migraine-like pain behaviors in mice. Cephalalgia 39, 111–122. 10.1177/0333102418779548, PMID: 29848111 PMC6081257

[ref31] Hawkins J. L. Moore N. J. Miley D. Durham P. L. (2018). Secondary traumatic stress increases expression of proteins implicated in peripheral and central sensitization of trigeminal neurons. Brain Res. 1687, 162–172. 10.1016/j.brainres.2018.03.003, PMID: 29522721 PMC5882570

[ref32] Hearing C. M. Chang W. C. Szuhany K. L. Deckersbach T. Nierenberg A. A. Sylvia L. G. (2016). Physical exercise for treatment of mood disorders: a critical review. Curr. Behav. Neurosci. Rep. 3, 350–359. 10.1007/s40473-016-0089-y, PMID: 28503402 PMC5423723

[ref33] International Classification of Headache Disorders (2018). Headache classification Committee of the International Headache Society (IHS) The international classification of headache disorders, 3rd Edn. Cephalalgia 38, 1–211. 10.1177/0333102417738202, PMID: 29368949

[ref34] Iyengar S. Johnson K. W. Ossipov M. H. Aurora S. K. (2019). CGRP and the trigeminal system in migraine. Headache 59, 659–681. 10.1111/head.13529, PMID: 30982963 PMC6593989

[ref35] Johnson D. Krenger W. (1992). Interactions of mast cells with the nervous system--recent advances. Neurochem. Res. 17, 939–951. 10.1007/BF00993271, PMID: 1357565

[ref36] Juhasz G. Zsombok T. Gonda X. Nagyne N. Modosne E. Bagdy G. (2007). Effects of autogenic training on nitroglycerin-induced headaches. Headache 47, 371–383. 10.1111/j.1526-4610.2006.00718.x, PMID: 17371354

[ref37] Julius D. Basbaum A. I. (2001). Molecular mechanisms of nociception. Nature 413, 203–210. 10.1038/35093019, PMID: 11557989

[ref38] Kaufmann D. Brennan K. C. (2018). The effects of chronic stress on migraine relevant phenotypes in male mice. Front. Cell. Neurosci. 12:294. 10.3389/fncel.2018.00294, PMID: 30283302 PMC6156251

[ref39] Kelman L. (2007). The triggers or precipitants of the acute migraine attack. Cephalalgia 27, 394–402. 10.1111/j.1468-2982.2007.01303.x, PMID: 17403039

[ref40] Kirschbaum C. Hellhammer D. H. (1989). Salivary cortisol in psychobiological research: an overview. Neuropsychobiology 22, 150–169. 2485862 10.1159/000118611

[ref41] Langford D. J. Bailey A. L. Chanda M. L. Clarke S. E. Drummond T. E. Echols S.. (2010). Coding of facial expressions of pain in the laboratory mouse. Nat. Methods 7, 447–449. 10.1038/nmeth.1455, PMID: 20453868

[ref42] Liu Y. Broman J. Edvinsson L. (2004). Central projections of sensory innervation of the rat superior sagittal sinus. Neuroscience 129, 431–437. 10.1016/j.neuroscience.2004.07.045, PMID: 15501600

[ref43] Markovics A. Kormos V. Gaszner B. Lashgarara A. Szoke E. Sandor K.. (2012). Pituitary adenylate cyclase-activating polypeptide plays a key role in nitroglycerol-induced trigeminovascular activation in mice. Neurobiol. Dis. 45, 633–644. 10.1016/j.nbd.2011.10.010, PMID: 22033344

[ref44] Mason B. N. Kaiser E. A. Kuburas A. Loomis M. M. Latham J. A. Garcia-Martinez L. F.. (2017). Induction of migraine-Like photophobic behavior in mice by both peripheral and central CGRP mechanisms. J. Neurosci. 37, 204–216. 10.1523/JNEUROSCI.2967-16.2016, PMID: 28053042 PMC5214631

[ref45] Massey E. W. (1982). Effort headache in runners. Headache 22, 99–100. 10.1111/j.1526-4610.1982.hed2203099.x, PMID: 7096076

[ref46] Miller A. Kitson G. Skalkoyannis B. Leach M. (2015). The effect of isoflurane anaesthesia and buprenorphine on the mouse grimace scale and behaviour in CBA and DBA/2 mice. Appl. Anim. Behav. Sci. 172, 58–62. 10.1016/j.applanim.2015.08.038, PMID: 26937061 PMC4768077

[ref47] Moskowitz M. Henrikson B. M. Markowitz S. Saito K. (1988). “Intra-and extracraniovascular nociceptive mechanisms and the pathogenesis of pain,” in Basic Mechanism of Headache. eds. Olesen J. Edvinsson L. (Amsterdam: Elsevier), 429–437.

[ref48] Nash J. M. Thebarge R. W. (2006). Understanding psychological stress, its biological processes, and impact on primary headache. Headache 46, 1377–1386. 10.1111/j.1526-4610.2006.00580.x, PMID: 17040334

[ref49] Nassini R. Materazzi S. Benemei S. Geppetti P. (2014). The TRPA1 channel in inflammatory and neuropathic pain and migraine. Rev. Physiol. Biochem. Pharmacol. 167, 1–43. 10.1007/112_2014_18, PMID: 24668446

[ref50] Nees T. A. Tappe-Theodor A. Sliwinski C. Motsch M. Rupp R. Kuner R.. (2016). Early-onset treadmill training reduces mechanical allodynia and modulates calcitonin gene-related peptide fiber density in lamina III/IV in a mouse model of spinal cord contusion injury. Pain 157, 687–697. 10.1097/j.pain.0000000000000422, PMID: 26588690

[ref51] Noseda R. Burstein R. (2013). Migraine pathophysiology: anatomy of the trigeminovascular pathway and associated neurological symptoms, CSD, sensitization and modulation of pain, Pain 154(Suppl. 1), S44–S53. 10.1016/j.pain.2013.07.021, PMID: 23891892 PMC3858400

[ref52] Ossovskaya V. S. Bunnett N. W. (2004). Protease-activated receptors: contribution to physiology and disease. Physiol. Rev. 84, 579–621. 10.1152/physrev.00028.2003, PMID: 15044683

[ref53] Patacchioli F. R. Monnazzi P. Simeoni S. De Filippis S. Salvatori E. Coloprisco G.. (2006). Salivary cortisol, dehydroepiandrosterone-sulphate (DHEA-S) and testosterone in women with chronic migraine. J. Headache Pain 7, 90–94. 10.1007/s10194-006-0274-6, PMID: 16575505 PMC3451699

[ref54] Peterson O. J. Cornelison L. E. Durham P. L. (2020). Neuroprotective effect of enriched chicken bone broth as a dietary supplement in a model of migraine mediated by early life stress. J. Med. Food 23, 1259–1265. 10.1089/jmf.2019.0312, PMID: 32326809 PMC7864107

[ref55] Pierce A. N. Di Silvestro E. R. Eller O. C. Wang R. Ryals J. M. Christianson J. A. (2016). Urinary bladder hypersensitivity and dysfunction in female mice following early life and adult stress. Brain Res. 1639, 58–73. 10.1016/j.brainres.2016.02.039, PMID: 26940840 PMC4870140

[ref56] Pierce A. N. Eller-Smith O. C. Christianson J. A. (2018). Voluntary wheel running attenuates urinary bladder hypersensitivity and dysfunction following neonatal maternal separation in female mice. Neurourol. Urodyn. 37, 1623–1632. 10.1002/nau.23530, PMID: 29464752 PMC6103918

[ref57] Pierce A. N. Ryals J. M. Wang R. Christianson J. A. (2014). Vaginal hypersensitivity and hypothalamic-pituitary-adrenal axis dysfunction as a result of neonatal maternal separation in female mice. Neuroscience 263, 216–230. 10.1016/j.neuroscience.2014.01.022, PMID: 24462609 PMC3962046

[ref58] Rainero I. Ferrero M. Rubino E. Valfre W. Pellegrino M. Arvat E.. (2006). Endocrine function is altered in chronic migraine patients with medication-overuse. Headache 46, 597–603. 10.1111/j.1526-4610.2006.00409.x, PMID: 16643554

[ref59] Recober A. Kaiser E. A. Kuburas A. Russo A. F. (2010). Induction of multiple photophobic behaviors in a transgenic mouse sensitized to CGRP. Neuropharmacology 58, 156–165. 10.1016/j.neuropharm.2009.07.009, PMID: 19607849 PMC2784010

[ref60] Rossi H. L. Lara O. Recober A. (2016). Female sex and obesity increase photophobic behavior in mice. Neuroscience 331, 99–108. 10.1016/j.neuroscience.2016.06.022, PMID: 27328418 PMC4947554

[ref61] Shyti R. Eikermann-Haerter K. Van Heiningen S. H. Meijer O. C. Ayata C. Joels M.. (2015). Stress hormone corticosterone enhances susceptibility to cortical spreading depression in familial hemiplegic migraine type 1 mutant mice. Exp. Neurol. 263, 214–220. 10.1016/j.expneurol.2014.10.015, PMID: 25447936

[ref62] Spierings E. L. Ranke A. H. Honkoop P. C. (2001). Precipitating and aggravating factors of migraine versus tension-type headache. Headache 41, 554–558. 10.1046/j.1526-4610.2001.041006554.x, PMID: 11437890

[ref63] Stucky N. L. Gregory E. Winter M. K. He Y. Y. Hamilton E. S. McCarson K. E.. (2011). Sex differences in behavior and expression of CGRP-related genes in a rodent model of chronic migraine. Headache 51, 674–692. 10.1111/j.1526-4610.2011.01882.x, PMID: 21521205 PMC4079043

[ref64] Theoharides T. C. Cochrane D. E. (2004). Critical role of mast cells in inflammatory diseases and the effect of acute stress. J. Neuroimmunol. 146, 1–12. 10.1016/j.jneuroim.2003.10.041, PMID: 14698841

[ref65] Theoharides T. C. Donelan J. Kandere-Grzybowska K. Konstantinidou A. (2005). The role of mast cells in migraine pathophysiology. Brain Res. Brain Res. Rev. 49, 65–76. 10.1016/j.brainresrev.2004.11.006, PMID: 15960987

[ref66] Theoharides T. C. Donelan J. M. Papadopoulou N. Cao J. Kempuraj D. Conti P. (2004). Mast cells as targets of corticotropin-releasing factor and related peptides. Trends Pharmacol. Sci. 25, 563–568. 10.1016/j.tips.2004.09.007, PMID: 15491778

[ref67] Theoharides T. C. Spanos C. Pang X. Alferes L. Ligris K. Letourneau R.. (1995). Stress-induced intracranial mast cell degranulation: a corticotropin-releasing hormone-mediated effect. Endocrinology 136, 5745–5750. 10.1210/endo.136.12.7588332, PMID: 7588332

[ref68] Toth L. A. (2015). The influence of the cage environment on rodent physiology and behavior: implications for reproducibility of pre-clinical rodent research. Exp. Neurol. 270, 72–77. 10.1016/j.expneurol.2015.04.010, PMID: 25913548

[ref69] Uddman R. Edvinsson L. Ekman R. Kingman T. McCulloch J. (1985). Innervation of the feline cerebral vasculature by nerve fibers containing calcitonin gene-related peptide: trigeminal origin and co-existence with substance P. Neurosci. Lett. 62, 131–136. 10.1016/0304-3940(85)90296-4, PMID: 2415882

[ref70] Varkey E. Cider A. Carlsson J. Linde M. (2009). A study to evaluate the feasibility of an aerobic exercise program in patients with migraine. Headache 49, 563–570. 10.1111/j.1526-4610.2008.01231.x, PMID: 18783448

[ref71] Varkey E. Cider A. Carlsson J. Linde M. (2011). Exercise as migraine prophylaxis: a randomized study using relaxation and topiramate as controls. Cephalalgia 31, 1428–1438. 10.1177/0333102411419681, PMID: 21890526 PMC3236524

[ref72] Wattiez A. S. Sowers L. P. Russo A. F. (2020). Calcitonin gene-related peptide (CGRP): role in migraine pathophysiology and therapeutic targeting. Expert Opin. Ther. Targets 24, 91–100. 10.1080/14728222.2020.1724285, PMID: 32003253 PMC7050542

[ref73] Wieseler J. Ellis A. Sprunger D. Brown K. McFadden A. Mahoney J.. (2010). A novel method for modeling facial allodynia associated with migraine in awake and freely moving rats. J. Neurosci. Methods 185, 236–245. 10.1016/j.jneumeth.2009.10.006, PMID: 19837113 PMC2814932

