# Supplementary figures and images for: Voluntary Wheel Running Partially Attenuates Early Life Stress-Induced Neuroimmune Measures in the Dura and Evoked Migraine-Like Behaviors in Female Mice
Source: Front Physiol. 2021 May 28;12:665732. doi: 10.3389/fphys.2021.665732 (PMC8194283; doi:10.3389/fphys.2021.665732)

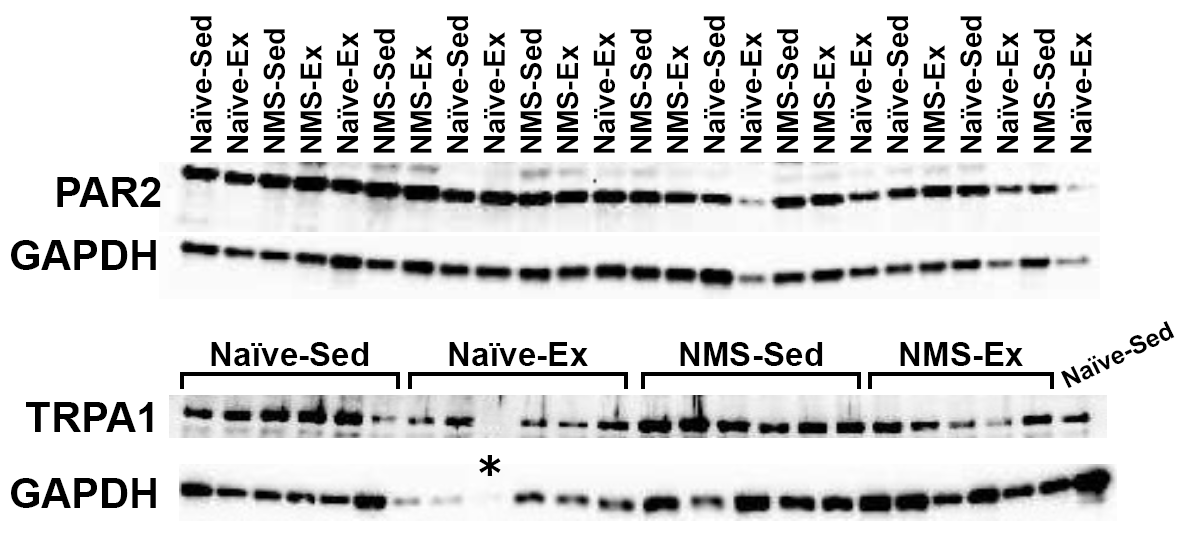

Supplement: Supplementary file 1 [file Image_1.TIF]
